# Anti-viral properties and mode of action of standardized *Echinacea purpurea *extract against highly pathogenic avian Influenza virus (H5N1, H7N7) and swine-origin H1N1 (S-OIV)

**DOI:** 10.1186/1743-422X-6-197

**Published:** 2009-11-13

**Authors:** Stephan Pleschka, Michael Stein, Roland Schoop, James B Hudson

**Affiliations:** 1 Institute for Medical Virology, Justus-Liebig-University Giessen, Frankfurterstr. 107, D-35392 Giessen, Germany; 2 Bioforce AG, Gruenaustr, CH-9325 Roggwil, Switzerland; 3 Department of Pathology & Laboratory Medicine, University of British Columbia, 2733 Heather Street, Vancouver V5Z 3J5, Canada

## Abstract

**Background:**

Influenza virus (IV) infections are a major threat to human welfare and animal health worldwide. Anti-viral therapy includes vaccines and a few anti-viral drugs. However vaccines are not always available in time, as demonstrated by the emergence of the new 2009 H1N1-type pandemic strain of swine origin (S-OIV) in April 2009, and the acquisition of resistance to neuraminidase inhibitors such as Tamiflu^® ^(oseltamivir) is a potential problem. Therefore the prospects for the control of IV by existing anti-viral drugs are limited. As an alternative approach to the common anti-virals we studied in more detail a commercial standardized extract of the widely used herb *Echinacea purpurea *(Echinaforce^®^, EF) in order to elucidate the nature of its anti-IV activity.

**Results:**

Human H1N1-type IV, highly pathogenic avian IV (HPAIV) of the H5- and H7-types, as well as swine origin IV (S-OIV, H1N1), were all inactivated in cell culture assays by the EF preparation at concentrations ranging from the recommended dose for oral consumption to several orders of magnitude lower. Detailed studies with the H5N1 HPAIV strain indicated that direct contact between EF and virus was required, prior to infection, in order to obtain maximum inhibition in virus replication. Hemagglutination assays showed that the extract inhibited the receptor binding activity of the virus, suggesting that the extract interferes with the viral entry into cells. In sequential passage studies under treatment in cell culture with the H5N1 virus no EF-resistant variants emerged, in contrast to Tamiflu^®^, which produced resistant viruses upon passaging. Furthermore, the Tamiflu^®^-resistant virus was just as susceptible to EF as the wild type virus.

**Conclusion:**

As a result of these investigations, we believe that this standard *Echinacea *preparation, used at the recommended dose for oral consumption, could be a useful, readily available and affordable addition to existing control options for IV replication and dissemination.

## Background

Influenza viruses (IV) continue to cause problems globally in humans and their livestock, particularly poultry and pigs, as a consequence of antigenic drift and shift, resulting frequently and unpredictably in novel mutant and re-assortant strains, some of which acquire the ability to cross species barriers and become pathogenic in their new hosts [1]. Prospects for the emergence of pandemic strains of swine and avian origin have been discussed in several recent reports [2,3]. Some of the highly pathogenic avian IV (HPAIV) strains, in particular H5N1, have occasionally infected humans and pose a severe threat because of their high pathogenicity, with mortality rates exceeding 60% [4,5].

The practicality and efficacy of control by timely vaccination has been questioned [1,6,7], and potential control of IV by synthetic anti-viral chemicals has usually been thwarted by the inevitable emergence of resistant strains, a situation that has been documented in the case of the M2 ion-channel inhibitors, such as adamantane derivatives, and the neuraminidase inhibitors such as oseltamivir and zanamivir [8,9]. Virus-strain specificity is another limitation in the use of these inhibitors.

Alternative approaches to therapy that overcome these obstacles are urgently needed and have been suggested. These include manipulation of specific signaling pathways known to be involved in virus replication [10,11]. As such, the Raf/MEK/ERK-signal transduction cascade and activation of the transcription factor NF-κB were shown to be essential for efficient nuclear export of the viral ribonucleoprotein (RNP) complexes. They have proven to be highly interesting targets, as their inhibition significantly reduces virus replication without emergence of resistant variants *in vitro *and *in vivo *[12-15]. Another approach is the use of broad-spectrum and chemically-standardized anti-IV herbal extracts and compounds with demonstrated efficacy *in vitro *[16-19]. These could conceivably afford a more generalized inhibition of all virus strains, either by virtue of inactivating the virus directly or by interfering with one or more essential stages in virus replication or dissemination. Furthermore anti-viral herbal extracts frequently exhibit multiple bioactivities [20], and this could enable their use at relatively low doses of the active compounds, possibly acting in synergy, while at the same time providing a relatively safe "drug" with few side effects. Needless to say, acquisition of resistance to herbal compounds is also a potential problem; consequently this would need to be evaluated, although if multiple bioactive compounds were involved, this would substantially reduce the risk of resistant viruses emerging.

We recently reported the anti-viral properties of a standardized preparation of *Echinacea purpurea *(Echinaforce^®^, EF), which has become a very popular herbal "remedy" for the symptoms of "colds and flu". In addition to possessing potent virucidal activity against several membrane containing viruses, including H3N2-type IV, at the recommended dose for oral consumption, the preparation also effectively reversed virus-induced pro-inflammatory responses in cultured epithelial cells [21]. Some *Echinacea*-derived preparations also possess selective anti-bacterial and immune modulation activities that might also contribute to their beneficial properties [22,23]. However, our studies also indicated that anti-viral and cytokine-inhibitory properties vary widely among different *Echinacea *species and components [24-26]; thus it is important to carry out research on *Echinacea *preparations that have been standardized and chemically characterized.

The objective of the present study was to investigate the anti-IV activity in more detail, and to elucidate possible mechanisms of action on a variety of IV strains (human and avian), with emphasis on a human isolate of the H5N1-type HPAIV, and to evaluate the potential for emergence of resistant strains, in comparison with oseltamivir (Tamiflu^®^).

## Results

### Echinaforce^® ^(EF) and Virus Concentration

**Figure 1:**
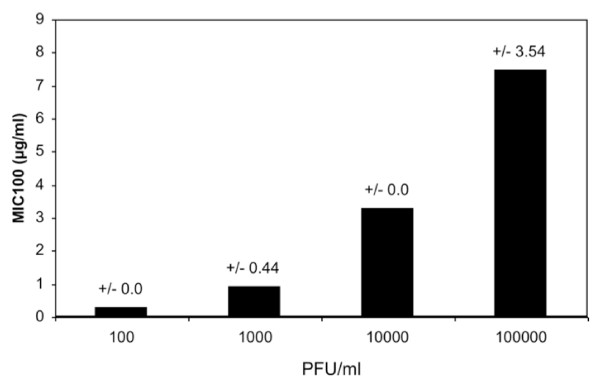
**MIC depends on the viral dose**. Increasing amounts of IV (Victoria, H3N2) were used to determine the MIC_100 _of EF. Serial dilutions of EF, in quadruplicate, were incubated with the amounts of IV indicated (10^2^, 10^3^, 10^4^, 10^5 ^PFU), and transferred to cells for CPE-endpoint determination, as described in Materials and Methods section. The MIC_100 _(μg/ml) is the concentration of EF that leads to complete prevention of CPE.

We reported previously that at concentrations up to 1.6 mg/ml (dry mass/vol, the recommended oral dose) the EF extract showed no apparent cytotoxic effects, according to trypan blue staining, MTT assays, or microscopic examination [[21], data not shown]. However at concentrations of >1.6 μg/ml ≥99% inactivation of H3N2-type IV was achieved (Table 1). The degree of inactivation depended on the virus dose, as might be expected (Fig. 1). MIC_100 _values increased from 0.32 μg/ml for 10^2 ^PFU/ml virus, up to 7.5 μg/ml for 10^5 ^PFU/ml.

**Table 1:** Anti-influenza virus (H3N2) effect of EF

EF dilution (μg/ml)	Virus titer (PFU, % of control)
1:30 (53.3)	< 0.1

1:10^2 ^(16)	< 0.1

1: 10^3 ^(1.6)	< 0.1

1: 10^4 ^(0.16)	1.0 ± 0

1: 10^5 ^(0.016)	110 ± 7.8

Aliquots of H3N2 virus, containing 10^5 ^PFU/ml, were incubated at 22°C for 60 min with the indicated concentrations of EF, and assayed for remaining PFU/ml

**Figure 2:**
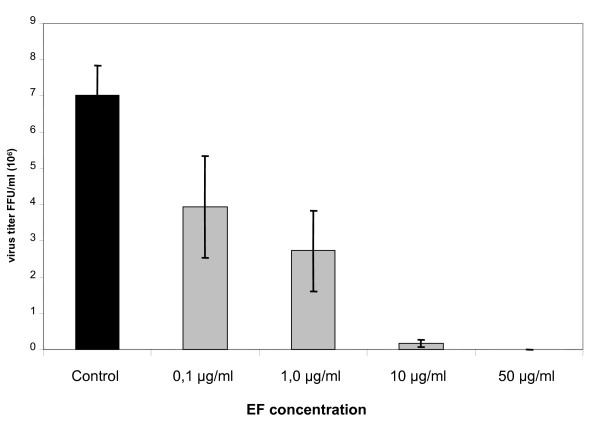
**EF acts in a dose dependent manner**. H5N1 HPAIV (MOI = 0.001) and MDCK cells were pre-incubated with EF at the indicated concentrations 1 hour prior to infection. Infected cells were then incubated in media with EF at the appropriate concentrations for 24 hours and the infectious titer was determined (FFU/ml). The experiment was performed in triplicate, and titrations in duplicate.

In order to exclude the possibility that the virucidal effect might be subtype specific or related only to human IV, we analyzed the effect of EF in non toxic concentrations on a human isolate of a H5N1-type HPAIV (KAN-1). Virus yield reduction assays were carried out with KAN-1, which had been pre-incubated with various concentrations of EF, from 0.1 to 50 μg/ml (Fig. 2). At the highest concentration the yield was reduced by more than 3 log_10_. Furthermore we tested the inhibitory effect of EF on human H1N1-type (PR8) and a H7-type HPAIV (FPV) and obtained comparable results (data not shown), indicating that EF affects not only human IV (H3N2, H1N1) but also both types (H5, H7) of HPAIV (data not shown).

### Time of addition of Echinaforce^®^

**Figure 3:**
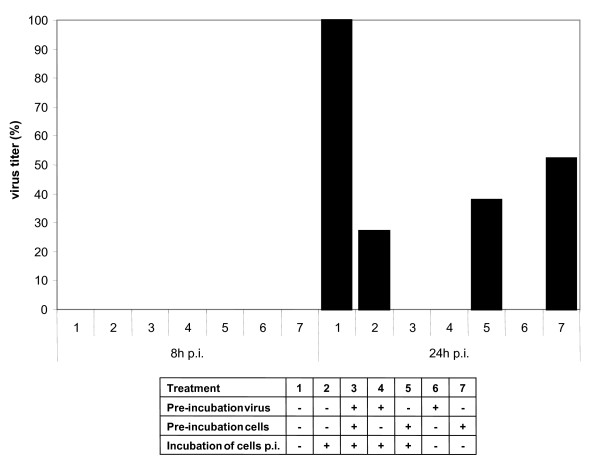
**Pre-treatment of IV with EF is most effective**. H5N1 HPAIV (MOI = 1) and MDCK cells were treated with EF (50 μg/ml) as indicated. Infected cells were then incubated in medium with or without EF for 8 and 24 hours and the infectious titer was determined (FFU/ml). The experiment was performed in triplicate, and titrations in duplicate.

All the IV strains tested, the human pathogenic Victoria (H3N2), PR8 (H1N1), S-OIV (H1N1), and the avian strains KAN-1 (H5N1) and FPV (H7N7) were susceptible to the EF, but only as a result of direct contact. Pre-incubation of cells with extract, followed by virus infection, or post-exposure of the infected cells to EF, inhibited virus replication to a lesser extent (data not shown). To investigate this in more detail, several experiments were performed with KAN-1 to determine the effect of adding EF at different times relative to virus infection of the cells. Complete inhibition was achieved by incubating KAN-1 and EF together before adding to the cells (Fig 3, lanes 3, 4, and 6). However other combinations of pre- and post-exposure to EF (lanes 2, 5, and 7) resulted in only partial reduction in virus production, compared to untreated (lane 1). These results suggest that EF was acting either directly on the virus or at a very early stage in the replication cycle. It is noteworthy to mention, that removal of EF containing medium 6.5 hours p.i. and further incubation in normal medium for 1.5 hours in order to prevent an exposure of newly formed virions to EF prior to titration, did not change this result.

### Intra-cellular RNP localization

**Figure 4:**
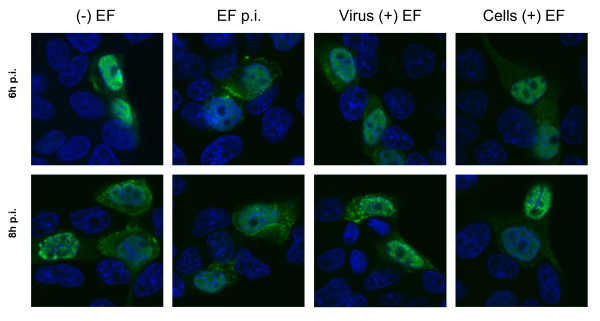
**Intra-cellular RNP production and localization is not affected by EF**. H5N1 HPAIV (MOI = 1) and MDCK cells were either left untreated or were treated with EF as follows: (-) EF, normal infection with no EF treatment; (EF p.i.), infected cells treated with EF (50 μg/ml) after infection; virus (+) EF, virus pretreated with EF (50 μg/ml); cells (+) EF, cells pretreated with EF (50 μg/ml), and infected with untreated virus. Infected cells were then incubated in medium with or without EF for 6 and 8 hours and the intra-cellular amount and localization of viral RNPs (green), as well as the nuclei (blue), were detected by immunofluorescence.

Next, the production and intra-cellular localization of viral RNP were determined by immunofluorescence, in MDCK cells infected with KAN-1, with and without EF treatment (Fig 4). In normally infected cells (- EF), the nucleocapsid protein (NP, green), which is the main component of the RNPs, appeared initially in the nucleus (6 hours) followed by migration to the cytoplasm (8 hours). The same pattern was seen in EF-treated cells infected with untreated virus (cells + EF), and in cells exposed to EF after infection (EF p.i.). However, when cells were infected with EF-treated virus (virus + EF), the overall number of positive cells was significantly reduced. Nevertheless, the amount and the localization of RNPs detected in cells infected with pre-treated IV was the same as for untreated cells infected with untreated virus. It should be noted that the treatment of infected cells at different time points p.i. did not affect the number of cells positive for NP staining (data not shown). These results suggest that EF affects a very early stage before replication, but once the virus has entered the cells its replication and spread are not affected.

### Interaction of Echinaforce^® ^with Viral HA

The first step in entry of IV into cells depends on the interaction between the viral HA and a specific cellular sialic acid containing receptor. If EF could inhibit this interaction by binding to the HA, then entry of virus might be prevented. Receptor binding of functional HA can be measured by its ability to agglutinate chicken erythrocytes, which can be easily enumerated visually. Direct interaction between virus and EF was therefore examined by inspecting viral hemagglutination (HA) activity in the presence and absence of EF. Results for the pandemic S-OIV (H1N1) and two HPAIV (H5, H7) are shown in Table 2. EF inhibited HA activity for all 3 virus strains, in a concentration and time-dependent manner. The same concentrations of EF without virus showed no hemagglutination, as expected (data not shown). In addition there was no visual evidence of erythrocyte lysis in any of the reactions. Therefore the inhibition in HA activity was due to an interference by EF. As this is effective against different human and avian strains, EF might exert an unspecific effect on IV replication by interfering with viral receptor binding and entry.

**Table 2:** Interaction of EF with viral HA

			μg/ml EF 1 hour					μg/ml EF 4 hours				
**IV ** **strain**	**Pos. ** **Ctrl.**	**Neg. ** **Ctrl.**	**50**	**100**	**200**	**400**	**800**	**50**	**100**	**200**	**400**	**800**

S-OIV (H1N1)	+	-	-	-	-	-	-	-	-	-	-	-

KAN-1 (H5N1)	+	-	+	+	+	-	-	-	-	-	-	-

FPV (H7N7)	+	-	+	+	+	-	-	-	-	-	-	-

+: indicates hemagglutinin activity (agglutination of erythrocytes)

-: indicates no hemagglutin activity. Ctrl, control

### Lack of Resistance to Echinaforce^®^

**Figure 5:**
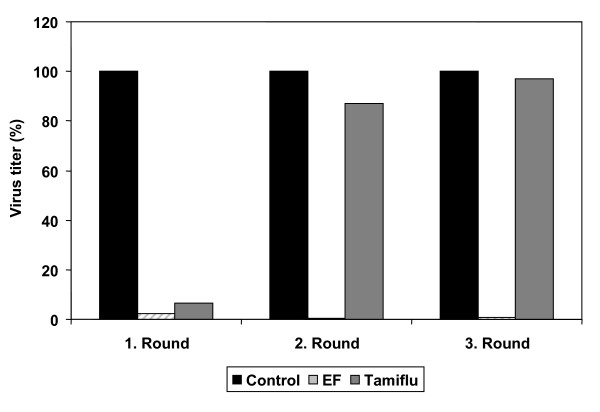
**EF treatment does not select for resistant IV variants**. MDCK cells were infected with KAN-1 (MOI = 0.001) and incubated 24 hours either with media without EF (black bars), or containing EF (50 μg/ml hatched bars) or Tamiflu^® ^(2 μM, grey bars). Supernatant was titrated by FFU assay and used for a second round of infection of fresh MDCK cells. Three passages (1st, 2nd, 3rd round) were performed and after each the virus titer (FFU/ml) was determined by FFU assay. FFU titres of EF- and Tamiflu^®^-treated samples were calculated as percentage of controls set at 100%. Shown is the mean of duplicate experiments titrated in duplicates.

Treatment with currently available anti-influenza drugs directly targeting the virus has the drawback that, due to the high mutation rate of IV, resistant strains will inevitably arise. This has been shown for neuraminidase inhibitors like Tamiflu^® ^in regard to seasonal IV, H5N1 HPAIV and in recent reports for the pandemic S-OIV [2,3,9,27,28]. Therefore, any competitive alternative should have the advantage of preventing emergence of resistant IV variants [11]. This might be different for a substance that unspecifically blocks virus activity. The possibility of emergence of EF-resistant virus was evaluated by comparing relative H5N1 virus yields in the presence and absence of EF, or Tamiflu^®^, during consecutive passages through cell cultures. Results are shown in Fig 5. After one round of replication virus yields were substantially reduced by 50 μg/ml EF or 2 μM Tamiflu^®^. However in rounds 2 and 3 the yields in the presence of Tamiflu^® ^were similar to controls, indicative of emergence of resistant virus variants, whereas in the presence of EF yields continually remained low, indicating lack of EF-resistant virus.

To determine if Tamiflu^®^-resistant virus remained sensitive to EF, the growth of Tamiflu^®^-resistant virus (produced in the above experiments) was tested in the presence and absence of EF. EF (50 μg/ml) reduced the yield of Tamiflu^®^-resistant virus by more than 3 log_10 _viral FFU, similar to that of standard virus (data not shown).

## Discussion

These results have shown that Echinaforce^® ^(EF), a standardized *Echinacea purpurea *extract, has potent anti-viral activity against all the IV strains tested, namely human Victoria (H3N2) and PR8 (H1N1), avian strains KAN-1 (H5N1) and FPV (H7N7), and the pandemic S-OIV (H1N1). Concentrations ranging from 1.6 mg/ml, the recommended dose for oral consumption, to as little as 1.6 μg/ml of the extract, a 1:1000 dilution, could inactivate more than 99% of virus infectivity, and treated virus gave rise to markedly reduced yields of virus in cell culture. However, direct contact between virus and EF was required for this inhibitory effect, since pre-treatment of cells before virus infection, or exposure of cells p.i. to EF, led to substantially less inhibition, indicating that the anti-viral effect was manifest at a very early stage in the infection process. This was then confirmed by the use of hemagglutination assays, which clearly showed that EF inhibited HA activity and consequently would block entry of treated virus into the cells. Nevertheless, the mechanism of this inhibition needs to be studied in more detail.

The general inhibition of EF against the different virus strains constitutes a significant advantage over other strain specific anti-virals, such as adamantanes [8,9]. Furthermore, the lack of emergence of EF-resistant viruses during sequential passage is a significant advantage over Tamiflu^®^, which under similar culture conditions readily allowed resistant virus strains to develop. In addition the Tamiflu^®^-resistant virus was still very sensitive to EF. These results indicate that EF could be helpful in IV control, and would be complemented by the known ability of EF to counteract pro-inflammatory cytokine and chemokine induction caused by IV and other viruses, as well as the selective anti-bacterial activities of *Echinacea *extracts [23]. Thus EF could play a multi-functional role during IV infections.

The Echinaforce^® ^extract contains known concentrations of potentially bioactive compounds [21,22], and these include the so-called standard markers such as phenolic caffeic acid derivatives, alkylamides, and polysaccharides, all of which have been proposed to be responsible for the purported medical benefits of various *Echinacea *species extracts [29]. However our recent studies on different types of *Echinacea *extract suggest that specific bioactivities may not be attributed to a single component. In addition EF, like other *Echinacea*-derived extracts, contains numerous other bioactive compounds such as flavonoids and alkaloids [29], and it is conceivable that the key to the relatively high potency of EF is the particular combination or balance of individual ingredients.

Recent studies on the Mediterranean herb *Cistus incanus *(rock rose) provide some interesting comparisons. Thus a polyphenol-rich *Cistus *extract showed similar anti-IV activities to those described in this report, suggesting a similar mode of action [18]. The mechanism of *Cistus *anti-viral activity was not elucidated however, so a comparative study of these two extracts could be useful and provide interesting implications for the design of effective anti-IV compounds.

In contrast, the study of Palamara et al [16] showed that an individual polyphenol, resveratrol, a common constituent of red grapes and various other plants, could inhibit IV replication by interfering with signaling pathways involved in viral RNP translocation. Thus an appropriate combination of plant polyphenols could provide a multifunctional approach to the control of influenza virus replication and its associated symptoms.

## Conclusion

The data presented in this work have shown that a standardized preparation of *Echinacae *has the potential to impair influenza virus propagation, including seasonal strains and strains of highly pathogenic avian influenza viruses as well as the new pandemic strain of swine origin at concentrations recommended for oral use and below. Furthermore the preparation does not induce emergence of resistant virus variants and is still active against strains that have become resistant to treatment with neuraminidase inhibitors. This potential, the availability and the lack of toxicity make this preparation an interesting option in the control and treatment of influenza virus infections

## Methods

### Standard *Echinacea *Preparation

Echinaforce^® ^(obtained from A. Vogel Bioforce AG, Roggwil, Switzerland) is a standardized preparation derived by ethanol extraction of freshly harvested *Echinacea purpurea *herb and roots (95:5). The composition of marker compounds (ie. those compounds known to characterize this species of *Echinacea*) was described previously [21]. The concentration of ethanol was 65% v/v. The final concentration of ethanol in the experimental reactions and cultures was too low to cause adverse effects on the cells or viruses. In addition the preparation was free of detectable endotoxin (as determined by means of a commercial assay kit, Lonza Walkersville Inc., MD, lower limit of detection 0.1 unit/ml), and the administered amount that was effective in our experiments, up to the recommended oral dose of 1.6 mg/ml, was not cytotoxic according to trypan blue staining, MTT formazan assays (MTT = 1-(4,5-dimethylthiazol-2-yl)-3,5-diphenylformazan), and microscopic examination [[21], and data not shown].

### Cell lines & Viruses

Madin-Darby canine kidney cells (MDCK) were acquired originally from ATCC and were passaged in Dulbecco's MEM (DMEM), in cell culture flasks, supplemented with 5-10% fetal bovine serum, at 37°C in a 5% CO_2 _atmosphere (cell culture reagents were obtained from Invitrogen, Ontario (CA) or Karlsruhe (DE)). No antibiotics or anti-mycotic agents were used for experiments performed in the Hudson laboratory. In the Pleschka laboratory the cell cultures were also grown in DMEM, 10% FCS but supplemented by 100 U/ml penicillin and 100 μg/ml streptomycin (P/S).

The following influenza A virus strains were used: human strain A/Victoria/3/75 (Victoria, H3N2) acquired from the BC Centre for Disease Control, Vancouver. The human HPAIV isolate A/Thailand/KAN-1/2004 (KAN-1, H5N1) was provided to S. Pleschka by P. Puthavathana, Thailand; the HPAIV A/FPV/Bratislava/79 (FPV, H7N7) and the human strain A/Puerto Rico/8/34 (PR8, H1N1) were obtained from the IV strain collection in Giessen, Germany; the human isolate of the 2009 pandemic IV of swine-origin A/Hamburg/1/09 (S-OIV, H1N1) was provided to S. Pleschka by M. Matrosovich, Marburg, Germany. KAN-1 and FPV or PR8 were propagated on MDCK cells with low serum but without trypsin (KAN-1, FPV) or in embryonated chicken eggs (PR8), respectively. All other strains were propagated on MDCK cells in the presence of trypsin (2.5 μg/ml). Stock viruses were prepared as clarified cell-free supernatants or allantois fluid, respectively, with titers ranging from 10^7 ^to 10^8 ^PFU (plaque-forming units) per ml. and stored at -75°C. Strains were titrated either by standard plaque assay or by focus forming assays (see below).

### MIC_100 _values

MIC_100 _values of EF were determined from CPE-endpoint assays, as follows: The *Echinacea *extract, in 200 μl aliquots, was serially diluted two-fold across replicate rows of a 96-well tray, in medium, starting at the recommended oral dose of 1.6 mg/ml. Virus, 100 PFU in 100 μl, was added to each well and allowed to interact with the extract for 60 min at 22°C. Following the incubation period, the mixtures were transferred to another tray of cells from which the medium had been aspirated. These trays were then incubated at 37°C, 5% CO_2 _until viral CPE were complete in control wells containing untreated virus (usually 2 days). Additional wells contained cells not exposed to virus. The MIC_100 _was the maximum dilution at which CPE was completely inhibited by the extract. In most assays the replicate rows gave identical end-points; when two-fold differences were encountered arithmetic means and standard deviations were calculated. In the alternative (intra-cellular) method, the cells were incubated with the diluted extracts first, before adding virus.

### Virus titrations

Strain Victoria (H3N2) was titrated by standard plaque assay techniques in MDCK cells with agarose overlays. The other strains were assayed by focus formation in MDCK cells as follows: Cells were grown overnight (to 90% confluency) in complete medium in 96-well trays, washed and inoculated with 50 μl of serially diluted (10^-1 ^to 10^-8^) virus in PBS containing 0.2% BA, 1 mM MgCl_2_, 0.9 mM CaCl_2_, 100 U/ml penicillin and 0.1 mg/ml streptomycin (PBS/BA), for 60 min at room temperature. The inoculum was replaced by 150 μl MC media (1× DMEM, BA, P/S, 1.5% methyl cellulose). Cells were incubated at 37°C, 5% CO_2 _for 44 hours. To detect foci of infection the cells were permeabilized with 330 μl fixing solution (4% paraformaldehyde, 1% triton X-100, in PBS) and stored at 4°C for 60 min followed by 3 washes with PBS/0.05% Tween 20, and incubation with 50 μl 1^st ^antibody (mouse anti-influenza A nucleoprotein mAb, BIOZOL BZL 10908) diluted in PBS/3% BA at room temperature for 60 min. Cells were then washed 3 × with PBS/Tween 20 and incubated with 2^nd ^antibody (anti-mouse HRP antibody Santa Cruz sc2005) diluted in PBS/3% BA at room temperature for 60 min. Finally cells were washed 3 × with PBS/Tween20 and incubated in 40 μl AEC staining solution (3-amino-9-ethylcarbazole, Sigma Chemical, AEC #101) for 60 min followed by washing in dH_2_O. Foci were scanned and analyzed by means of Photoshop software (Adobe). All titrations were performed in duplicate.

### Pre-incubations

In some experiments aliquots of virus (H3N2 or H5N1) in PBS/BA or the cells in complete medium, were pre-incubated with EF (50 μg/ml) at room temperature or 37°C respectively for 60 min, prior to infection. Infected cells and controls were then incubated in medium containing EF (50 μg/ml) at 37°C, 5% CO_2 _for 24 hours, at which time supernatants were removed for focus assays.

### Intra-cellular RNP localization

Cells were grown and infected on cover slips, and pre-incubations of the viruses were carried out as described above. Cells were fixed, at different times post infection (p.i.), washed with PBS and incubated with 1^st ^antibody, as described above (2.4). Incubation with 2^nd ^antibody (rabbit anti-mouse Texas red) diluted in PBS/3%BA was carried out at room temperature for 60 min in the dark. Cells were washed again and incubated with DAPI (0.1 mg/ml PBS/3%BA, Roth Germany) for 10 min in the dark to stain nuclei. After further washing the cover slips with cells were covered with Moviol + DABCO (Moviol, Aldrich, glycerine, Merck, ddH2O, Tris-Cl pH 8.5 + 1,4-Diazobicyclo [2.2.2]octane, Merck) on glass slides. Cells were examined and digitized with a TCS SP5 confocal laser scanning microscope (Leica, Germany).

### Hemagglutination assay

25 μl EF in PBS at the indicated concentrations were added to wells of a 96-well tray. Thereafter 25 μl of virus with ca. 2560 HAU/ml were added. The plates were incubated for 60 min at 4°C. After this incubation period, 50 μl of chicken erythrocyte suspension (CES, 0.5% in PBS) were added to each well. The plates were further incubated for 60 min or 4 hours at 4°C. Wells were visually inspected for the presence or absence of hemagglutination. Positive and negative controls without EF treatment or without virus were included. To assay possible hemagglutination by EF itself, 50 μl of EF in PBS at the indicated concentrations were incubated with 50 μl CES for 60 min or 4 hours at 4°C. All assays were performed in quadruplicate.

### Virus Resistance Assay

MDCK cells grown over night at 37°C and 5% CO_2 _were pre-incubated with 2 ml complete medium (1× DMEM, 10% FCS, Pen/Strep) with or without EF (50 μg/ml), at 37°C and 5% CO_2 _for 60 min. In parallel virus in PBS/BA was incubated with EF (50 μg/ml) or left untreated for 60 min. After the pre-incubation period the cells were washed and infected with 500 μl virus suspension (MOI = 0,001) (+/-) Echinaforce^® ^(50 μg/ml). Cells were then incubated for 60 min in the dark at room temperature after which the inoculum was removed. Cells were further incubated in 2 ml medium (DMEM/BA/P/S with Echinaforce^® ^(50 μg/ml), Tamiflu^® ^(2 μM) or without test substances) at 37°C, 5% CO_2 _for 24 hours. Samples of the supernatants were collected, which were then assayed by focus forming assay for further determination of infectious virus. Following the assays, these supernatants were used to infect another set of cultures under the same conditions as described above. This process of sequential infection with supernatants was repeated once more to yield in total three rounds of infection and replication. Experiments done in duplicates were stopped when the Tamiflu^® ^sample reached titers of the untreated control.

### Biosafety

All experiments with infectious virus were performed according to German and Canadian regulations for the propagation of influenza A viruses. All experiments involving highly pathogenic influenza A viruses and the pandemic S-OIV were performed in a biosafety level 3 (BSL3) containment laboratory approved for such use by the local authorities (RP, Giessen, Germany).

## Abbreviations

CPE: cytopathic effects; EF: Echinaforce^®^; FFU: focus-forming unit; HA: hemagglutinin; HAU: hemagglutinating units; IV: influenza virus; PFU: plaque-forming unit; RNP: (viral) ribo-nucleoprotein; S-OIV: swine-origin influenza virus.

## Competing interests

The work was in part financially supported by Bioforce AG (to S.P and J.H.). There were no competing interests.

## Authors' contributions

SP directed and participated in the studies on avian and H1N1 viruses, and co-wrote the manuscript.

MS carried out the experimental work in Germany.

RS organized the overall project, supplied the standardized source material, and helped edit the manuscript.

JH carried out the experimental work in Canada, and co-wrote and edited the manuscript.

## References

[B1] Cannell JJ Zasloff M Garland CF Scragg R Giovannucci E On the epidemiology of influenza Virology J 2008 5 29 10.1186/1743-422X-5-29 PMC2279112 18298852

[B2] Michaelis M Doerr HW Cinatl J Novel swine-origin influenza A virus in humans: another pandemic knocking at the door Med Microbiol Immunol 2009 198 175 183 10.1007/s00430-009-0118-5 19543913

[B3] Neumann G Noda T Kawaoka Y Emergence and pandemic potential of swine-origin H1N1 influenza virus Nature 2009 459 931 939 10.1038/nature08157 19525932 PMC2873852

[B4] Bahlky H Avian influenza: The tip of the iceberg Ann Thorac Med 2009 3 154 157 10.4103/1817-1737.43085 PMC2700449 19561900

[B5] Suzuki Y The Highly Pathogenic Avian Influenza H5N1-Initial Molecular Signals for the Next Influenza Pandemic Chang Gung Med J 2009 32 258 263 19527604

[B6] Hayden F Developing New Antiviral Agents for Influenza Treatment: What does the Future Hold? Clin Infec Dis 2009 48 S3 S13 10.1086/591851 19067613

[B7] Jefferson T Di Pietrantonj C Debalini MG Rivetti A Demicheli V Inactivated influenza vaccines: methods, policies, and politics J Clin Epidemiol 2009 62 677 686 10.1016/j.jclinepi.2008.07.001 19124222

[B8] Lackenby A Thompson CI Democratis J The potential impact of neuraminidase inhibitor resistant influenza Curr Op Infec Dis 2008 21 626 638 10.1097/QCO.0b013e3283199797 18978531

[B9] Cheng PKC Leung TWC Ho ECM Leung PKC Ng AYY Lai MYY Lim WWL Oseltamivir- and Amantadine-Resistant Influenza viruses A (H1N1) Emerg Infec Dis 2009 15 966 968 10.3201/eid1506.081357 PMC2727327 19523305

[B10] Ludwig S Planz O Pleschka S Wolff T Influenza-virus-induced signaling cascades: targets for antiviral therapy? Trends Mol Med 2003 9 46 52 10.1016/S1471-4914(02)00010-2 12615037

[B11] Ludwig S Targeting cell signaling pathways to fight the flu: towards a paradigm change in anti-influenza therapy J Antimic Ther 2009 64 1 4 10.1093/jac/dkp161 19420020

[B12] Pleschka S Wolff T Ehrhardt C Hobom G Planz O Rapp UR Ludwig S Influenza virus propagation is impaired upon specific inhibition of the Raf/MEK/ERK signaling cascade Nat Cell Biol 2001 3 301 305 10.1038/35060098 11231581

[B13] Ludwig S Wolff T Ehrhardt C Wurzer WJ Reinhardt J Planz O Pleschka S MEK inhibition impairs influenza B virus propagation without emergence of resistant variants FEBS Lett 2004 561 37 43 10.1016/S0014-5793(04)00108-5 15013748

[B14] Wurzer WJ Erhardt C Pleschka S Berberich-Seibelt F Wolff T Walczak H Planz O Ludwig S NF-kappaB-dependent of tumor necrosis factor-related apoptosis-inducing ligand (TRAIL) and Fas/FasL is crucial for efficient influenza virus propagation J Biol Chem 2004 279 30931 30937 10.1074/jbc.M403258200 15143063

[B15] Mazur I Wurzer WJ Ehrhardt C Pleschka S Puthavathana P Silberzahn T Wolff T Planz O Ludwig S Acetylsalicylic acid (ASA) blocks influenza virus propagation via its NF-kappaB-inhibiting activity Cell Microbiol 2007 9 1683 94 10.1111/j.1462-5822.2007.00902.x 17324159

[B16] Palamara AT Nencioni L Aquilano K De Chiara G Hernandez L Cozzolino F Ciriolo MR Garaci E Inhibition of Influenza Virus Replication by Resveratrol J Infec Dis 2005 191 1719 1729 10.1086/429694 15838800

[B17] Wang X Jia W Zhao A Wang X Anti-influenza Agents from Plants and Traditional Chinese Medicine Phytother Res 20 335 341 10.1002/ptr.1892 16619359

[B18] Ehrhardt C Hrincius ER Korte V Mazur I Droebner K Poetter A Dreschers S Schmolke M Planz O Ludwig S A Polyphenol rich plant extract, CYSTUS052, exerts anti influenza virus activity in cell culture without toxic side effects or the tendency to induce viral resistance Antivir Res 2007 76 38 47 10.1016/j.antiviral.2007.05.002 17572513

[B19] Droebner K Ehrhardt C Poetter A Ludwig S Planz O CYSTUS052, a polyphenol-rich plant extract, exerts anti-influenza virus activity in mice Antivir Res 2007 76 1 10 10.1016/j.antiviral.2007.04.001 17573133

[B20] Hudson JB Towers GHN Phytomedicines as antivirals Drugs of the Future 1999 24 295 320 10.1358/dof.1999.024.03.858620

[B21] Sharma M Anderson SA Schoop R Hudson JB Induction of pro-inflammatory cytokines by respiratory viruses and reversal by standardized *Echinacea*, a potent antiviral herbal extract Antiviral Res 2009 83 165 170 10.1016/j.antiviral.2009.04.009 19409931

[B22] Sharma M Vohra S Arnason JT Hudson JB *Echinacea *Extracts Contain Significant and Selective Activities Against Human Pathogenic Bacteria Pharmac Biol 2008 46 111 116 10.1080/13880200701734919

[B23] Vohra S Adams D Hudson JB Moore JA Vimalanathan S Sharma M Burt A Lamont E Lacaze N Arnason JT Lee TD Selection of Natural Health Products for Clinical Trials: a Preclinical Template Can J Physiol Pharmacol 2009 87 371 378 10.1139/Y09-021 19448735

[B24] Hudson J Vimalanathan S Kang L Treyvaud Amiguet V Livesey J Arnason JT Characterization of antiviral activities in *Echinacea *root preparations Pharmac Biol 2005 43 790 796 10.1080/13880200500408491

[B25] Vimalanathan S Kang L Treyvaud Amiguet V Livesey J Arnason JT Hudson J *Echinacea purpurea *aerial parts contain multiple antiviral compounds Pharmac Biol 2005 43 740 745 10.1080/13880200500406354

[B26] Vimalanathan S Arnason JT Hudson JB Anti-inflammatory activities of *Echinacea *extracts do not correlate with traditional marker components Pharmac Biol 2009 47 430 435 10.1080/13880200902800204

[B27] Hurt AC Emergence and spread of oseltamivir-resistant A(H1N1) influenza viruses in Oceania, South East Asia and South Africa Antivir Res 2009 83 90 93 10.1016/j.antiviral.2009.03.003 19501261

[B28] Morbidity Mortality Weekly Report Oseltamivir-Resistant Novel Influenza A (H1N1) Virus Infection in Two Immunosuppressed Patients-Seattle Washington MMWR 2009 58 32 893 896 19696719

[B29] Barnes J Anderson LA Gibbons S Phillipson JD *Echinacea *species (*Echinacea angustifolia *(DC.) Hell. *Echinacea pallida *(Nutt.) Nutt., *Echinacea purpurea *(L.) Moench: a review of their chemistry, pharmacology and clinical properties J Pharm Pharmacol 2005 57 929 954 10.1211/0022357056127 16102249

